# Case Report: Long-term TCR repertoire dynamics in a disease-free survival stage IIIB-N3 lung adenocarcinoma patient treated with anti-PD-1 followed by surgery

**DOI:** 10.3389/fimmu.2026.1836904

**Published:** 2026-07-27

**Authors:** Xiaoqian Zhai, Yongcheng Liu, Manhua Wang, Zhenkun Liu, Kaili Huang, Xuexue Wu, Mingyu Fan, Yan Huang, Xinxia Gu, Jie Liu, Xuyu Cai, Ye Wang, Lili Jiang, Daxing Zhu

**Affiliations:** 1 Department of Medical Oncology, Cancer Center, West China Hospital, Sichuan University, Chengdu, Sichuan, China; 2 Lung Cancer Center, West China Hospital, Sichuan University, Chengdu, Sichuan, China; 3 Institute of Thoracic Oncology, West China Hospital, Sichuan University, Chengdu, China; 4 Frontiers Science Center for Disease-related Molecular Networks, West China Hospital, Sichuan University, Chengdu, Sichuan, China; 5 West China School of Medicine, Sichuan University, Chengdu, Sichuan, China; 6 Department of Thoracic Surgery, West China Hospital, Sichuan University, Chengdu, Sichuan, China; 7 Department of Pathology of West China Hospital, Sichuan University, Chengdu, China; 8 Laboratory of Infectious Diseases and Vaccine, West China School of Medicine, West China Hospital, Sichuan University, Chengdu, Sichuan, China; 9 Department of Respiratory and Critical Care Medicine, West China Hospital of Sichuan University, Chengdu, Sichuan, China

**Keywords:** case report, non-small cell lung cancer (NSCLC), PD-1, stage IIIB-N3, T-cell receptor (TCR)

## Abstract

Immune checkpoint blockade (ICB) therapy has dramatically improved the survival outcomes of patients with locally advanced unresectable and metastatic non-small cell lung cancer (NSCLC). In particular, accumulated clinical trials and systematic reviews have further verified that induction ICB regimen following surgery confers superior benefits for IIIB-N3 NSCLC. Herein, we present a clinical case of a patient diagnosed with bulky contralateral mediastinal N3 (cT1bN3M0) left upper lung adenocarcinoma and devoid of actionable driver gene alterations. The patient achieved pathological complete response following anti-PD-1 treatment and subsequently underwent left upper lobectomy, followed by anti-PD-1 therapy for three consecutive years. To date, the patient has maintained a recurrence-free survival status for more than 5 years. Notably, we dynamically monitored the T-cell receptor (TCR) repertoire throughout the entire treatment course. Further analysis of TCR profiles revealed distinct dynamic patterns of certain specific T-cell clonotypes during treatment. These clonal alterations may help interpret sustained immune responses and warrant further exploration as candidate indicators for therapeutic outcome.

## Introduction

The advent of immune checkpoint blockade (ICB) has profoundly reshaped the survival prospects of patients with locally advanced unresectable and metastatic non-small cell lung cancer (NSCLC) (1, 2). During immune responses, the T-cell receptor (TCR) generally consists of α and β chains, which govern its specificity for intercellular immune interactions (3). In clinical treatment, anti-PD-1/PD-L1 agents bind to PD-1 or PD-L1 molecules. This process enables TCRs to recognize tumor antigens presented in the form of antigen–MHC complexes, thereby activating effector T cells and eliciting potent antitumor immune responses in immunotherapy (4, 5).

For patients with unresectable stage IIIB-N3 NSCLC, the National Comprehensive Cancer Network recommends concurrent radical chemoradiotherapy and follow-up with immunotherapy. Nevertheless, data from the PACIFIC trial revealed that patients with stage IIIB-N3 NSCLC achieve a median progression-free survival (PFS) of merely 16.8 months (6). Accordingly, improving PFS and overall survival remains an urgent clinical research priority for this patient population. Accumulated clinical trials and systematic reviews have further verified that ICB regimen confers superior event-free survival and PFS benefits for IIIB-N3 NSCLC (7–9).

Current evidence confirms that TCR diversity in peripheral blood and tumor tissues is closely correlated with clinical prognosis and immunotherapeutic efficacy (10, 11). Accordingly, exploring dynamic alterations in TCR diversity and identifying core functional clonotypes is of great clinical value for optimizing immunotherapy stratification. In order to understand immune response persistence and explore possible prognostic indicators, we report a driver-negative stage IIIB-N3 lung adenocarcinoma patient who achieved pathological complete response (pCR) after induction anti-PD-1 therapy followed by radical resection. Serial TCR sequencing at multiple key time points was performed to characterize immune repertoire dynamics, aiming to provide preliminary evidence for understanding immune response persistence and exploring possible prognostic indicators.

## Method

### Samples and genomic DNA extraction

Before chemotherapy and immunotherapy, tumor samples were obtained by percutaneous biopsy of the left upper lung lesion. PD-L1 expression was assessed using the 22C3 pharmDx assay, and targeted next-generation sequencing was performed using the OncoD-C1021B panel. Key actionable alterations in *EGFR*, *ALK*, and *ROS1* were not detected (**Supplementary Table 1**). During video-assisted thoracoscopic left upper lobectomy, the resected primary tumor, station 4R lymph node, and matched adjacent normal tissue were defined as Surg-resected T, Surg-resected LN, and Surg-resected N, respectively. Peripheral blood mononuclear cell (PBMC) samples were collected at four time points: before ICB treatment, before surgery, and at 1 and 3 years after surgery during adjuvant ICB therapy, and were designated as Pre-ICB-PBMC, Pre-Surgery-PBMC, adjuv-ICB-1-year-PBMC, and adjuv-ICB-3-year-PBMC, respectively. Written informed consent was obtained from the patient.

For TCRβ sequencing, genomic DNA was extracted from PBMCs using the QIAamp DNA Blood Mini Kit and from formalin-fixed paraffin-embedded (FFPE) tissue sections using the QIAamp DNA FFPE Tissue Kit (Qiagen, Hilden, Germany), according to the manufacturer’s protocols. At least 300 ng of gDNA from PBMCs and 500 ng of gDNA from FFPE tissues were used for library preparation.

### TCRβ CDR3 immunosequencing and depth normalization

The CDR3 region of the human TCRβ chain was amplified using a two-round multiplex polymerase chain reaction (PCR) strategy. Briefly, a primer pool containing Vβ forward and Jβ reverse primers was used to amplify TCRβ V(D)J rearrangements, followed by a second PCR with universal primers for library construction. Libraries were sequenced on the Illumina HiSeq 4000 platform.

To reduce bias from unequal sequencing depth, all samples were randomly downsampled to 6,000,000 raw reads before alignment using seqtk v1.4-r122 (https://github.com/lh3/seqtk). The lowest-yield sample, Pre-Surgery-PBMC, generated 6,407,191 raw reads, confirming that all samples exceeded this threshold. Per-sample raw reads, productive reads, and numbers of unique productive clonotypes are provided in **Supplementary Table 2**.

### Clonotype identification and repertoire analysis

CDR3β sequences were identified using MiXCR v4.3.2 (12). Reads were aligned with mixcr align, clonotypes were assembled with mixcr assemble, and TRB clones were exported with mixcr exportClones --chains TRB. A clonotype was defined at the amino acid level as a unique productive CDR3β sequence. Non-productive clonotypes, including out-of-frame sequences and sequences containing premature stop codons, were excluded from downstream analyses.

Repertoire diversity, overlap, and clonotype tracking were analyzed using immunarch v0.10.3 (https://immunomind.github.io/docs/). Diversity analysis was performed using repDiversity, clonotype tracking was carried out using trackClonotypes, and pairwise repertoire overlap was accomplished using repOverlap. Hierarchical clustering and heatmap visualization were performed using pheatmap v1.0.12 (https://CRAN.R-project.org/package=pheatmap). Cross-sample comparisons were based on relative clonotype frequencies and clonotype presence/absence after depth normalization, rather than absolute read counts, to reduce the influence of sequencing depth and tissue composition.

### Definition of enriched clonotypes

A CDR3β clonotype was defined as enriched in Sample B relative to Sample A if it met both criteria: (i) relative frequency >0.01% in Sample B; and (ii) at least a fourfold increase in frequency in Sample B compared with Sample A. Clonotypes undetected in Sample A were considered enriched only when they met the abundance threshold in Sample B.

## Results

### The ICB treatment efficacy in a patient with unresectable IIIB-N3 NSCLC

**Figure 1:**
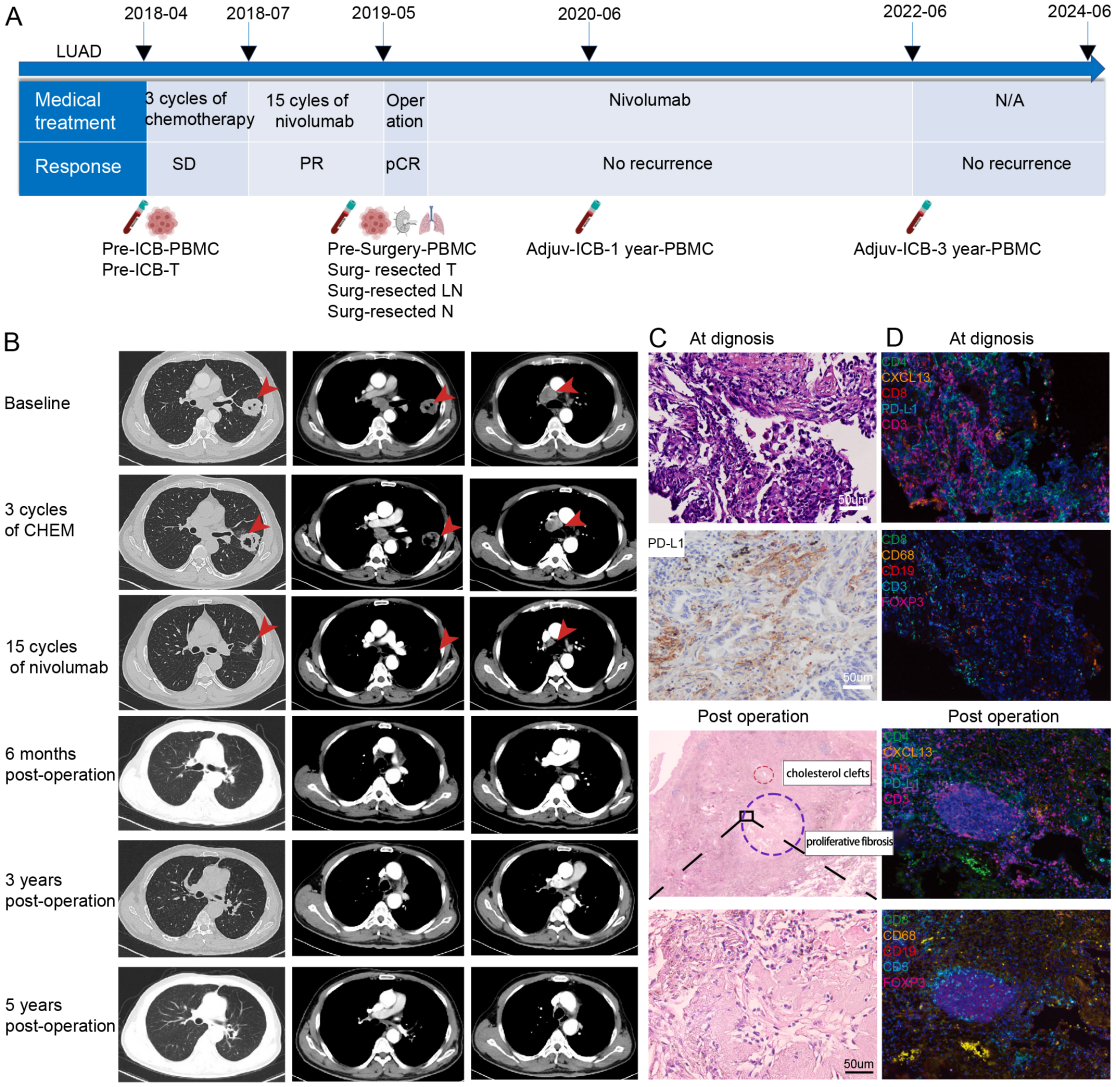
Clinical characteristic of the patient. **(A)** Timeline of clinical course including treatment response and sample collection time. **(B)** Representative chest CT images of the patient during the treatment process; the red arrow indicates the tumor. **(C)** Representative H&E images at diagnosis and post-operation. **(D)** Representative multiple immunofluorescence images of the patient showing tertiary lymphoid structures at diagnosis and post-operation. LUAD, lung adenocarcinoma; CHEM, chemotherapy; ICB, immune checkpoint blockade; Adjuv, adjuvant; PBMC, peripheral blood mononuclear cell; T, tumor; LN, lymph node; N, normal lung.

In April 2018, a 60-year-old male patient underwent pretreatment baseline CT, which revealed a 4.0 × 3.6 cm left upper lobe primary tumor and an enlarged contralateral pretracheal retrocaval (2R/4R) mediastinal lymph node with short-axis diameters of 3.5 × 2.8 cm. These baseline CT findings indicated N3 stage metastasis (**Figure 1B**; **Supplementary Figure 1**). No metastatic lesions were detected on chest, abdominal, cranial CT, and bone scintigraphy [single-photon emission computed tomography (SPECT)]. Based on the above imaging results, the patient was clinically staged as cT1bN3M0, stage IIIB left upper lobe lung adenocarcinoma with massive contralateral mediastinal lymph node metastasis. Genetic detection confirmed negative alterations in *EGFR*, *ALK*, and *ROS1* genes. The positive expression rate of PD-L1 was approximately 70%, and the tumor mutation burden reached 31.0 mutations/Mb. From 8 May to 11 July 2018, the patient received three cycles of chemotherapy at our institution, with Pemetrexed 900 mg and Carboplatin 500 mg on day 1 of each cycle. Subsequent chest computed tomography revealed only mild tumor regression after treatment. Given the unsatisfactory efficacy of chemotherapy and the high PD-L1 expression level in this patient, the multidisciplinary team discussion reached a consensus that additional chemoradiation would provide limited clinical benefit for this patient’s lesion and immunotherapy was expected to yield prominent clinical benefits. Thus, we determined to administer immunotherapy for the patient. After he received 15 times Opdivo 200 mg q2w monotherapy from 6 August 2018 to 12 April 2019, the efficacy was evaluated as partial response (PR) (**Figures 1A–C**). Further therapeutic regimens were formulated through multidisciplinary team consultation. Given the remarkable clinical benefit derived from immunotherapy, the patient underwent video-assisted thoracoscopic left upper lobectomy combined with bilateral mediastinal lymph node dissection in May 2019, followed by comprehensive pathological sampling of the entire tumor bed. Postoperative pathological results confirmed pCR, with no viable tumor cells detected in the primary lesion and resected lymph nodes. Histological manifestations mainly included proliferative fibrosis, neovascularization, cholesterol clefts, and diffuse inflammatory cell infiltration (**Figure 1C**). Notably, tertiary lymphoid structures were identified in postoperative specimens after immunotherapy, accompanied by abundant infiltration of CD3^+^CD8^+^ T cells (**Figure 1D**). From May 2019 to May 2022, the patient received adjuvant nivolumab maintenance therapy for three consecutive years after surgery. Circulating tumor DNA sequencing performed at 1 and 3 years lasted 3 years from June 2020 to June 2022, which postoperatively verified sustained molecular complete response. Meanwhile, TCR sequencing was also performed on peripheral blood samples collected at the same two time points, which were obtained from the patient receiving neoadjuvant ICB therapy at 1 and 3 years postoperatively, namely, adjuv-ICB-1 year-PBMC and adjuv-ICB-3 year-PBMC. Up to the time of manuscript preparation, the patient had remained free of tumor recurrence for more than 5 years.

### TCR characteristics further suggested potential molecular mechanisms of immunotherapy

**Figure 2:**
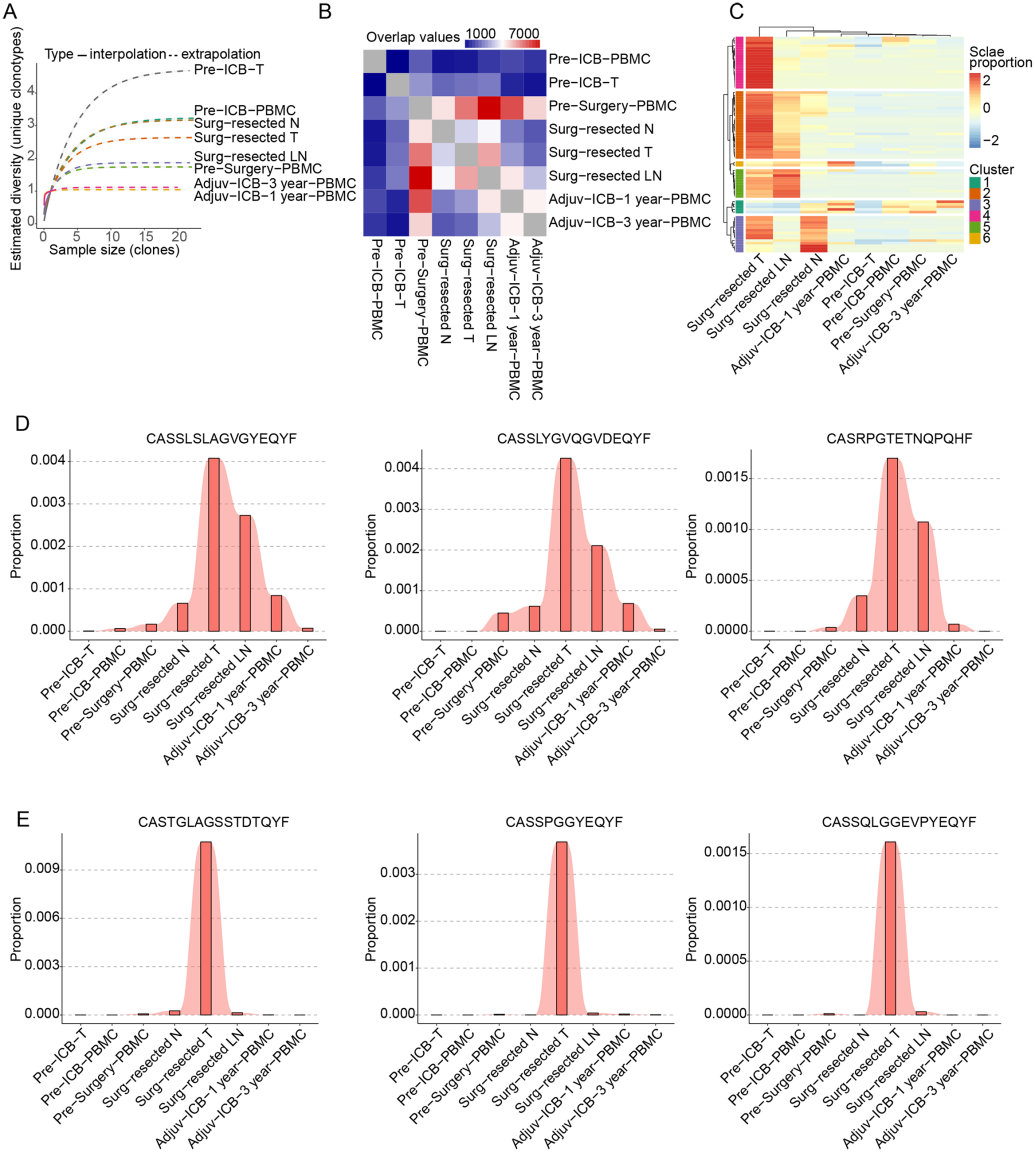
TCR sequencing results of the patient. **(A)** Rarefaction curves estimating unique TCRβ clonotypes across treatment stages. **(B)** Heatmap of the repertoire overlap analysis of the patient during different treatment stages. Red: High level of overlap values. Blue: Low level of overlap values. The higher overlap value indicated that a large number of clones were shared between the two samples and *vice versa*. **(C)** Heatmap of TCR clonotypes enrichment clustered by the mRNA expression characteristics of the patient during different treatment stages. **(D)** The TCR clonotypes that were enriched in the following patient stages: Surg-resected T, Surg-resected LN, and Adjuv-ICB-1-year-PBMC. **(E)** The TCR clonotypes that were enriched in the Surg-resected T stage of the patient. TCR, T cell receptor; ICB, immune checkpoint blockade; PBMC, peripheral blood mononuclear cell; Surg, surgery; Adjuv, adjuvant; T, tumor; LN, lymph node; N, normal lung.

Furthermore, we dynamically profiled TCR repertoire characteristics throughout the treatment course to elucidate the underlying mechanisms of therapeutic response. Dynamic TCR sequencing included PBMCs of pre-ICB (Pre-ICB-PBMC), pre-surgery (Pre-Surgery-PBMC), neoadjuvant ICB therapy after 1 and 3 years of surgery (adjuv-ICB-3 year-PBMC, adjuv-ICB-3 year-PBMC), and the pre-ICB tumor (Pre-ICB-T), surgery-resected tumor (Surg-resected T), surgery-resected normal tissue (Surg-resected N), and surgery-resected lymph node (Surg-resected LN) harvested from right mediastinal station 4R. Rarefaction-based analysis suggested a reduction in observed TCRβ clonotype richness in the surgically resected tumor bed after immunotherapy and in PBMC samples collected at 1 and 3 years after adjuvant ICB therapy compared with baseline levels (**Figure 2A**). This pattern was interpreted descriptively as treatment-associated clonal focusing rather than as a definitive decrease in all dimensions of global TCR repertoire diversity. To determine the origin of the expanded clonotypes, we compared the 100 most abundant TCRβ clonotypes in the Surg-resected T and Pre-Surgery-PBMC samples with the Pre-ICB-T sample. Notably, 99 of the top 100 clonotypes in the Surg-resected T and 85 in the Pre-Surgery-PBMC were already detectable pre-treatment, indicating that the dominant expanded clonotypes predominantly originated from pre-existing repertoires rather than newly generated clones (**Supplementary Figures 2A, B**; **Supplementary Table 3**). Repertoire overlap analysis showed that Pre-ICB-T, Surg-resected T, and 1-year post-treatment PBMC shared a relatively large number of TCRβ clonotypes (**Figure 2B**). This finding may suggest persistence or recirculation of related T-cell clonotypes during treatment, although functional validation would be required to determine their antigen specificity and biological roles. In addition, comparative analysis of TCR profiles in Pre-ICB-T and Surg-resected-T identified distinct characteristics of enriched TCR clones. We further classified these enriched TCRβ clonotypes into six clusters based on their longitudinal abundance patterns, among which clusters 2, 4, and 5 were markedly expanded in both Surg-resected T and Surg-resected LN (**Figure 2C**). Similarly, in the analysis of Pre-Surgery-PBMC and Pre-ICB-T, cluster 6 showed enrichment in both Surg-resected T and Surg-resected LN samples (**Supplementary Figure 2C**). Specifically, three representative clonotypes, namely, CASSLSLAGVGYEQYF, CASSLYGVQGVDEQYF, and CASRPGTENQPQHF clonotypes, were upregulated in both tumors and PBMC after 1 year of therapy, whereas CASTGLAGSSTDTQYF, CASSPGGYEQYF, and CASSQLGGEVPYEQYF were exclusively enriched within tumor lesions (**Figures 2D, E**).

To precisely track the fate of pre-existing low-frequency clones, we identified clonotypes with a baseline relative abundance below 0.0001 (supported by more than 10 reads). Longitudinal clustering analysis revealed that cluster 5 underwent substantial expansion and enriched in tumor and lymph node samples after ICB treatment (**Supplementary Figures 3A, B**). Subsequent intersection analysis of post-treatment expanded and enriched clonotypes with low-frequency clones within cluster 5 identified 11 overlapping TCRβ clonotypes (**Supplementary Figures 3C, D**).

## Discussion

Herein, we reported a clinical case of stage IIIB-N3 left upper lung adenocarcinoma that attained pCR after ICB followed with radical surgery. The patient recovered well postoperatively, and postoperative pathology confirmed pCR. During the 5-year follow-up, there were no signs of recurrence, demonstrating good therapeutic effects. In the field of locally advanced NSCLC treatment, induction immunotherapy followed by surgery has been extensively studied, with several reports documenting obvious clinical responses of downstaging in patients with locally advanced disease similar to our case (13, 14). This case further validates that patients with initially unresectable N3 NSCLC may achieve tumor downstaging after immunotherapy, thereby gaining access to curative surgery, which could improve their long-term prognosis.

Furthermore, longitudinal dynamic monitoring of TCR repertoires in this case may provide preliminary clues regarding sustained antitumor immune responses followed by anti-PD-1 therapy. Our rarefaction-based analysis suggested a reduction in observed TCRβ clonotype richness in the surgically resected tumor bed after immunotherapy, with a similar reduction observed in PBMC samples collected at 1 and 3 years after adjuvant ICB therapy compared with baseline levels (**Figure 2A**). This finding should be interpreted as a descriptive indication of clonal focusing at an equal sequencing depth rather than as a definitive decrease in all aspects of global TCR repertoire diversity. Notably, no obvious difference in rarefaction-based observed clonotype richness was observed between the 1-year and 3-year postoperative PBMC samples during immune maintenance therapy (**Figure 2A**). Relevant studies have concluded that the long-term stability of TCR diversity reflects the maintenance of immune function and may exert a predictive value for long-term prognosis (15, 16). Our preliminary observation suggests a potential similarity in the immune repertoire characteristics at these two time points, which may imply sustained treatment-induced immune responses, and may partially contribute to the prolonged recurrence-free survival seen in this patient. However, limited by the sample size, it does not allow us to draw definitive conclusions regarding treatment efficacy, and further investigation in larger cohorts is required to validate this finding.

Some studies indicated that clinical outcomes of patients receiving ICB are closely correlated with specific TCR clonotypes (17, 18), implying that these clonotypes may offer clues relevant to immunotherapy response and merit further investigation as potential candidate indicators for treatment effect. As shown in **Figure 2D**, PBMCs at both 1 and 3 years after adjuvant ICB therapy remained detectable at considerable proportions in CASSLSLAGVGYEQYF, CASSLYGVQGVDEQYF, and CASRPGTENQPQHF clonotypes. We preliminarily speculate that these clonotypes may be associated with durable treatment-associated immune reactivity against tumor cells. In contrast, CASTGLAGSSTDTQYF, CASSPGGYEQYF, and CASSQLGGEVPYEQYF showed a notable increase only in tumor tissues (**Figure 2E**), reflecting unique distribution features of these T-cell subsets in the tumor microenvironment. Their potential relevance to clinical prognosis deserves further investigation. Meanwhile, our longitudinal tracking and intersection analyses confirm that at least part of the dominant post-treatment functional clonotypes originated directly from rare, pre-existing tumor-infiltrating clones.

To explore the potential antigen specificity of the expanded TCR repertoire, we performed TCRMatch analysis on expanded or newly detected CDR3β clonotypes after ICB treatment. Several clonotypes showed high similarity to TCRs recognizing viral epitopes, including SARS-CoV-2 and influenza epitopes, whereas no high-confidence matches to common tumor-associated antigens were identified in current public databases (**Supplementary Table 4**). Thus, these results should be interpreted cautiously, as the expanded clonotypes may represent cross-reactive or bystander T-cell populations rather than tumor-specific responses. These observations are preliminary and require further validation in future studies. Despite the fact that notable changes in distinct TCR clonotypes were observed in this study, the exact T-cell subsets these clones belong to, their biological features, and underlying regulatory mechanisms still remain poorly understood. Further validation using patient-specific neoantigen prediction, peptide-MHC multimer assays, and single-cell TCR/transcriptomic analyses is warranted. Different TCR diversity metrics capture distinct aspects of repertoire structure. Therefore, the rarefaction-based changes observed in this single-patient longitudinal case should be interpreted cautiously, given differences in the sampling method, tissue composition, T-cell density, and treatment-induced pathological changes, and should be validated in larger matched cohorts with functional analyses (19–22).

## Conclusion

We herein report an unresectable N3-stage NSCLC patient who achieved satisfactory clinical benefits from ICB and subsequently underwent curative surgical resection. Our results revealed a rarefaction-based reduction in observed TCRβ clonotype richness following immunotherapy relative to baseline levels, accompanied by the persistence and expansion of selected clonotypes. Several dominant TCR clonotypes persisted in peripheral blood samples at 1 and 3 years following adjuvant immunotherapy, showing stable clonal dynamics during long-term follow-up. Meanwhile, other distinct TCR clonotypes were found to be specifically enriched within tumor tissues, suggesting their potential involvement in local antitumor immune responses.

## Data Availability

The data presented in the study are deposited in the National Center for Biotechnology Information (NCBI) Sequence Read Archive (SRA) repository, accession number PRJNA1466079.

## Supplementary material

Supplementary Figure 1 Imaging changes and surgical specimen findings of left lung lesion and mediastinal 2R/4R lymph nodes. **(A)** CT characteristics before chemotherapy, red arrows indicate mediastinal lymph nodes. **(B)** CT characteristics after PD-L1 therapy, red arrows indicate mediastinal lymph nodes. **(C)** Resected 2R/4R lymph nodes at mediastinal stations.

Supplementary Figure 2 Pre-existing TCR clonotypes dominated the highly expanded repertoire after ICB treatment. **(A)** Detection and expansion status of the 100 most abundant TCR clonotypes in the Surg-resected T sample compared with the Pre-ICB-T sample; **(B)** Detection and expansion status of the 100 most abundant TCR clonotypes in the Pre-Surgery-PBMC sample compared with the Pre-ICB-T sample; **(C)** Longitudinal abundance patterns of TCR clonotypes that were expanded or newly detected in the Pre-Surgery-PBMC sample.

Supplementary Figure 3 Longitudinal dynamics of low-frequency pre-existing tumor-infiltrating TCR clonotypes. **(A)** Clustering of low-frequency Pre-ICB-T TCR clonotypes according to their longitudinal abundance patterns; **(B)** Representative highly expanded Cluster 5 clonotypes in the Surg-resected T sample; **(C)** Venn diagram showing the overlap between post-treatment tumor/lymph-node-enriched clonotypes and low-frequency Pre-ICB-T Cluster 5 clonotypes; **(D)** Longitudinal abundance patterns of the 11 overlapping clonotypes.
